# Supplementary material for: CAR T-Cell Targeting of Macrophage Colony-Stimulating Factor Receptor
Source: Cells. 2022 Jul 13;11(14):2190. doi: 10.3390/cells11142190 (PMC9323367; doi:10.3390/cells11142190)
Supplement: Supplementary file 1 [file cells-11-02190-s001.zip › cells-1800387-supplementary.pdf]

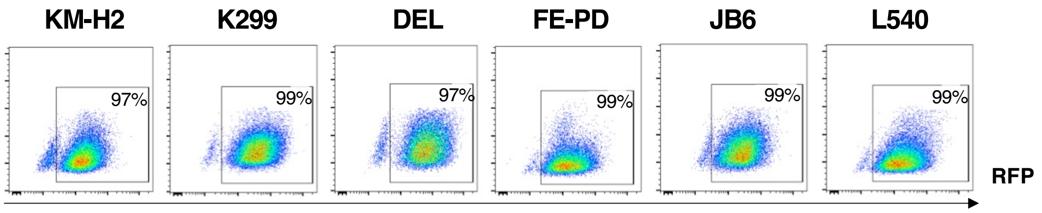

Figure S1 Flow sorting of firefly luciferase and red fluorescent protein-expressing lymphoma cells. The indicated Hodgkin's lymphoma (KM-H2, L540) or anaplastic large cell lymphoma cell lines (K299, DEL, FE-PD, JB6) were transduced with the SFG ffLuc/RFP retroviral vector and were purified by flow sorting for RFP expression. Flow cytometric analysis for RFP expression is shown.

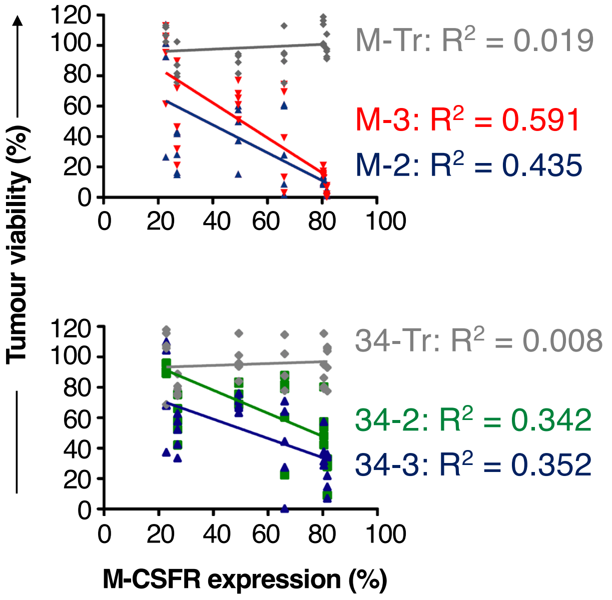

Figure S2. Correlation between cytolytic activity of M-CSFR-specific CAR T-cells and M-CSFR expression. CAR-engineered T-cells were co-cultured with Hodgkin's lymphoma (KM-H2, L540) or anaplastic large cell lymphoma cell lines (K299, DEL, FE-PD, JB6) for 72 hours, before cancer cell viability was quantified by luciferase assay. Graphs plot percentage tumour cell viability against percentage cell surface M-CSFR expression on each lymphoma cell line, as determined by flow cytometry. Data were obtained from a minimum of five independent experiments and each dot represents an individual sample measured for lymphoma cell viability. Correlation analysis was performed using simple linear regression analysis.

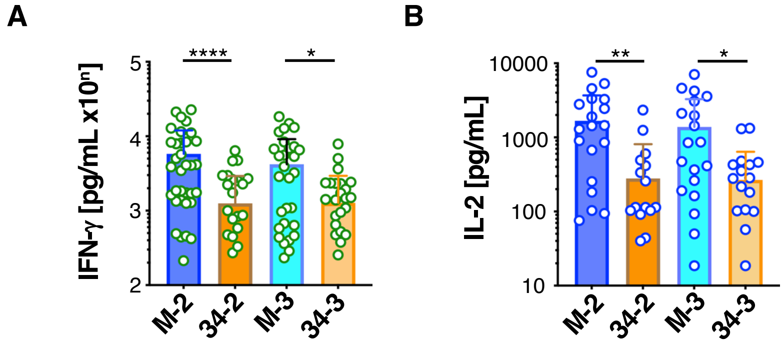

Figure S3. Pooled analysis of cytokine release by M-CSFR-specific CAR T-cells when cultured with lymphoma cells. Production of IFN-γ **(A)** and IL-2 **(B)** by M-CSF and IL-34 containing 2G and 3G CAR T-cells has been pooled across all six Hodgkin's lymphoma (KM-H2, L540) or anaplastic large cell lymphoma cell lines (K299, DEL, FE-PD, JB6). Statistical analysis was performed using one-way ANOVA and significant differences are indicated.
****$p<0.0001$; ***$p<0.001$; ** $p<0.01$; * $p<0.05$.
